# DDBJ Database updates and computational infrastructure enhancement

**DOI:** 10.1093/nar/gkz982

**Published:** 2019-11-14

**Authors:** Osamu Ogasawara, Yuichi Kodama, Jun Mashima, Takehide Kosuge, Takatomo Fujisawa

**Affiliations:** The Bioinformation and DDBJ Center, National Institute of Genetics, Mishima, Shizuoka, 411-8540, Japan

## Abstract

The Bioinformation and DDBJ Center (https://www.ddbj.nig.ac.jp) in the National Institute of Genetics (NIG) maintains a primary nucleotide sequence database as a member of the International Nucleotide Sequence Database Collaboration (INSDC) in partnership with the US National Center for Biotechnology Information and the European Bioinformatics Institute. The NIG operates the NIG supercomputer as a computational basis for the construction of DDBJ databases and as a large-scale computational resource for Japanese biologists and medical researchers. In order to accommodate the rapidly growing amount of deoxyribonucleic acid (DNA) nucleotide sequence data, NIG replaced its supercomputer system, which is designed for big data analysis of genome data, in early 2019. The new system is equipped with 30 PB of DNA data archiving storage; large-scale parallel distributed file systems (13.8 PB in total) and 1.1 PFLOPS computation nodes and graphics processing units (GPUs). Moreover, as a starting point of developing multi-cloud infrastructure of bioinformatics, we have also installed an automatic file transfer system that allows users to prevent data lock-in and to achieve cost/performance balance by exploiting the most suitable environment from among the supercomputer and public clouds for different workloads.

## INTRODUCTION

The DNA Data Bank of Japan (DDBJ) (https://www.ddbj.nig.ac.jp) (1) is a public database of nucleotide sequences established at the National Institute of Genetics (NIG) (https://www.nig.ac.jp/nig). Since 1987, the DDBJ Center has been collecting annotated nucleotide sequences as its traditional database service. This endeavour has been conducted in collaboration with GenBank (2) at the US National Center for Biotechnology Information (NCBI) and in partnership with the European Nucleotide Archive (ENA) (3) at the European Bioinformatics Institute (EBI). The collaborative framework is called the International Nucleotide Sequence Database Collaboration (INSDC) (4), and the product database from this framework is called the International Nucleotide Sequence Database (INSD).

Within the INSDC framework, the DDBJ Center also services the DDBJ Sequence Read Archive (DRA) for raw sequencing data and alignment information from high-throughput sequencing platforms (5), the BioProject for sequencing project metadata, and BioSample for sample information (1,6). This comprehensive resource of nucleotide sequences and associated biological information complies with the INSDC policy that guarantees free and unrestricted access to data archives (7). In addition to these INSDC databases, the DDBJ Center has accepted functional genomics experiments in the Genomic Expression Archive (GEA) which is counterpart of the Gene Expression Omnibus at NCBI (8) and the ArrayExpress at EBI (9). For human individual genotype and phenotype data requiring authorized access, the DDBJ Center has provided the controlled-access database Japanese Genotype-phenotype Archive (JGA) in collaboration with the National Bioscience Database Center (NBDC) in the Japan Science and Technology Agency (JST) since 2013 (10).

The supercomputer system operated by the NIG as a computational infrastructure for developing the DDBJ databases is also provided for use as large-scale computational resources to Japanese researchers in the fields of medicine and biology (11). In early 2019, the NIG supercomputer system was replaced in order to accommodate the recent rapid growth of the genome data archives.

In the present article, we report on updates to the abovementioned services at the DDBJ Center, and on the new supercomputer system. All of the resources described here are available from https://www.ddbj.nig.ac.jp, and most of the archival data can be downloaded at ftp://ftp.ddbj.nig.ac.jp.

## DDBJ ARCHIVAL DATABASE UPDATES

### Data contents: unrestricted- and controlled-access databases

The DDBJ has traditionally accepted nucleotide sequences with annotations and has released them in flat-file format. From June 2018 to May 2019, the traditional DDBJ database accepted 6330 nucleotide data submissions consisting of 9 760 101 entries, most of which were made by Japanese research groups (4835 submissions; 76.4%). The DDBJ has periodically released whole traditional data from the INSD, including both conventional sequence data and bulk sequence data such as whole-genome shotgun (WGS), transcriptome shotgun assembly (TSA), and targeted locus study (TLS) data, four times per year. However, because we were in the process of substantially upgrading our supercomputer system, we skipped generating the DDBJ periodical normally scheduled for release on March 2019. During the period from June 2018 to May 2019, DDBJ periodical releases in the form of number of entries increased from 532 382 985 to 2 097 223 144, and from 1 466 817 057 639 to 5 261 978 280 583 in the number of base pairs.

In the periodical release 116, DDBJ contributions to the INSD amounted to 3.80% of the entries and 3.38% of the total base pairs. A detailed statistical breakdown of the number of records is shown on the DDBJ website (https://www.ddbj.nig.ac.jp/stats/release-e.html#total_data). Note in the periodical release 116, many of bulk sequence data are lacking, because (i) very large-scale sequence data came from GenBank and ENA and (ii) DDBJ has not yet adopted the new format used for accession numbers (for information about the new format, see https://www.ddbj.nig.ac.jp/activities/icm-reports-e.html#2018).

In the period between June 2017 and May 2018, 44 118 runs of high-throughput sequencing data were registered to the DRA. As of 12 September 2019, the DRA has distributed 4.0 PB of sequencing data in the SRA (2.9 PB) and FASTQ (1.1 PB) formats. However, due to a shortage of available storage space, NCB/EBI SRA data was suspended from April 2017 to May 2019. After the storage space expansion, we resumed mirroring in June 2019. The GEA has archived 31 functional genomics experiments, and the data of 15 experiments are available via file transfer protocol (FTP) at the GEA database website (ftp://ftp.ddbj.nig.ac.jp/ddbj_database/gea). The FANTOM6 consortium Capped Analysis of Gene Expression (CAGE) data, which quantified transcriptomic profiles in human dermal fibroblasts after suppressing 285 long non-coding RNAs (12), are available under the accession numbers ‘E-GEAD-312’ and ‘E-GEAD-313’. The GEA metadata are searchable at the integrated index service for public gene expression datasets ‘All of gene expression’ (AOE) of the Database Center for Life Science (DBCLS) (13).

The JGA is a controlled-access database for genotype and phenotype data of human individuals (10) like the database of Genotypes and Phenotypes (dbGaP) at NCBI (14) and the European Genome-phenome Archive (EGA) at EBI (15). As of 12 September 2019, the JGA has archived 179 studies, 251 161 samples and 307 TB of individual-level human datasets submitted by Japanese researchers. The archived file size has tripled in one year due primarily to an increase in the number of whole-genome sequencing data submissions. The summaries of 118 studies are available to the public both on the JGA (https://ddbj.nig.ac.jp/jga/viewer/view/studies) and the NBDC (https://humandbs.biosciencedbc.jp/en/data-use/all-researches) websites. To access individual-level data of these public studies, users are required to submit data usage requests to the NBDC (https://humandbs.biosciencedbc.jp/en/data-use). The DDBJ Center has provided the AMED Genome group sharing Database (AGD), on which private genome data are shared among restricted users (a paid service). In early 2019, the DDBJ Center has implemented the Global Alliance for Genomics and Health (GA4GH) molecular beacon (16) to AGD for searching specific variants in the registered-access manner.

In collaboration with EBI, the GEA and JGA metadata will be indexed by the Omics Discovery Index (OmicsDI) at EBI (17) to enhance discoverability of the omics datasets.

## THE NIG SUPERCOMPUTER

The supercomputer system operated by the NIG provides indispensable computational resources and storages for development and operation of entire DDBJ databases and for Japanese researchers who require large-scale computing platform especially in the fields of medicine and biology (11).

In order to accommodate the current increase of the INSD data, we began upgrading the NIG supercomputer system in late 2017. Prior to the main system upgrade of the NIG supercomputer, we introduced new storage systems to migrate the DNA database archives. The building of the main system commenced in late 2018 and it became available to researchers in March 2019.

### The computing system

**Figure 1.**
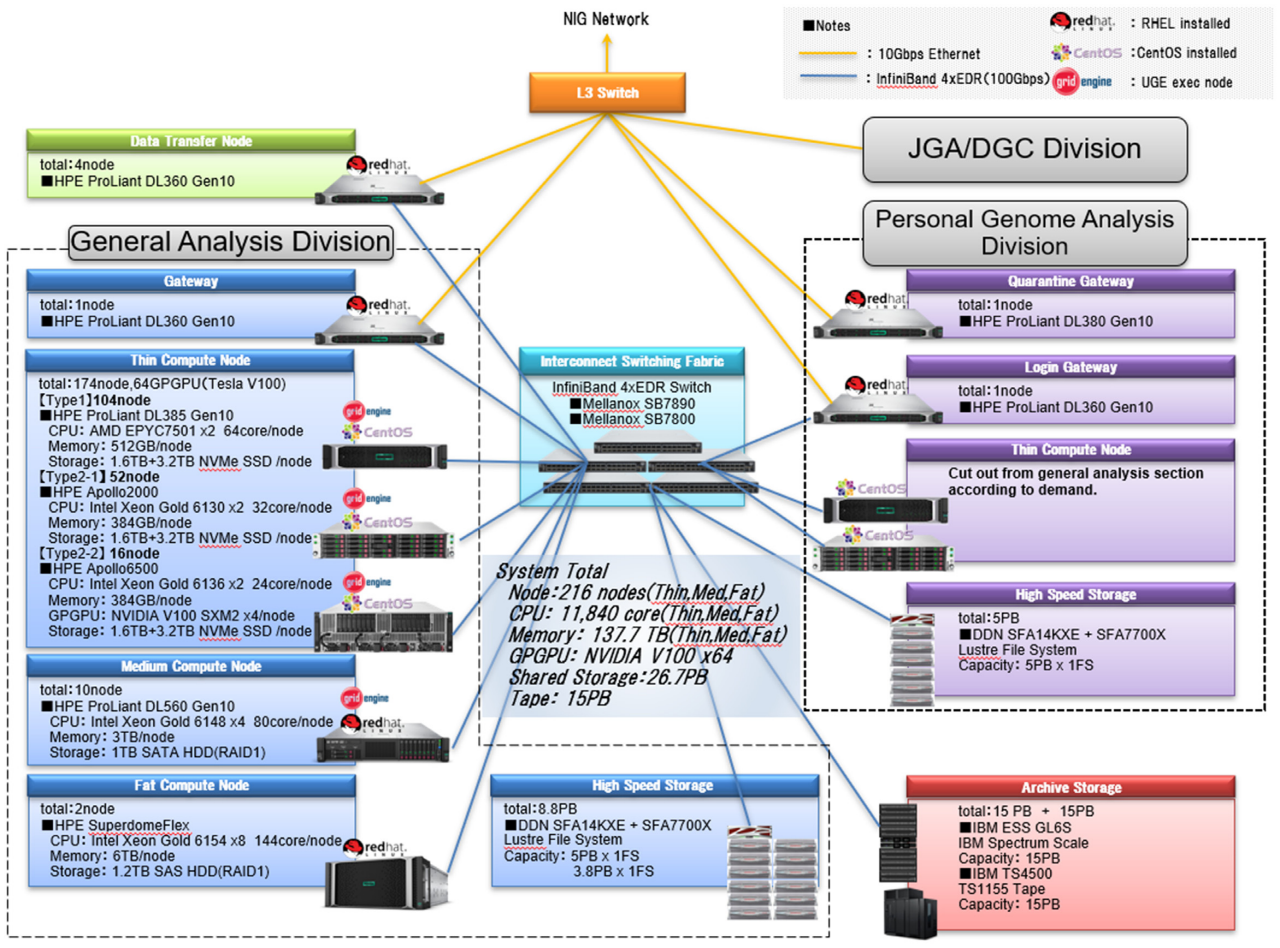
General architecture of the NIG supercomputer installed in 2019. Based on the previous system, the NIG Supercomputer 2019 mainly consists of a distributed memory HPC cluster, high-performance parallel distributed file systems for calculation, and large capacity archiving storage systems for the DNA database. Those systems are interconnected via a high-throughput low-latency network (InfiniBand) and various management networks (Ethernet).

The design goal of the NIG supercomputer 2019 is to provide both high-performance computing (HPC) and big data analysis platforms especially suitable for large-scale genome analysis (Figure 1). The principal parameters of the NIG supercomputer are as follows: The peak performance is 1.1 PFLOPS (CPU: 599.8 TFLOPS, GPU 499.2 TFLOPS), the total memory capacity is 138.8 TB, and the total storage capacity is 43.8 PB.

The NIG supercomputer 2019 includes two types of computing systems: a distributed memory HPC cluster (for general purpose), and non-uniform memory access (NUMA)-based large-scale shared memory calculation nodes (for de novo assembly or other memory-intensive calculations) (18–20).

The HPC cluster includes 16 GPU nodes which have four GPUs (NVIDIA Tesla V100 SXM2) for each chassis, which allow genome analysis tools including GATK4 (21) and Mutect2 (22) to accelerate more than one order, by using dedicated analysis system (e.g. Parabricks genome pipeline https://www.parabricks.com/).

The system is connected to the Internet with the bandwidth of 30 Gbps via the SINET5 network system hosted by National Institute of Informatics (NII), Japan (23). For long-distance transfer of large-scale genome data, our system is equipped with an Aspera server of 10 Gbps total bandwidth.

### The storage system

As described above, the NIG supercomputer provides both a high-performance computational infrastructure and a comprehensive DNA database in one system. To achieve these, there are two storage systems in the NIG supercomputer: the storage area for the calculation suitable for I/O intensive tasks (Lustre file system, 13.8 PB in total) and the storage area for the DNA data archive, constructed as a hierarchical storage system with 15 PB hard disk drive (HDD)-based storage and 15 PB tape library system (24–27).

In addition to those large-scale storage areas, each thin calculation node described above has 4.8 TB of NVMe SSD as its local storage space. Those storage systems can be used not only for simple local storage but also for constructing an on-demand Lustre file system (Ihara,S. and Deshmukh,R. Lustre On Demand: Evolution of Data Tiering on Storage System., DDN storage. https://www.eofs.eu/_media/events/lad18/08_rahul_deshmukh_lad18_lustre_on_demand_si_rd_final2.pdf) that works in a coordinated manner with task scheduling systems such as a Univa Grid Engine (UGE) (http://www.univa.com).

### Personal genome analysis system

Requisitions for the use of HPC systems have been increasing with the advent of application studies in medical research fields. The DDBJ Center has been constructing the JGA with the cooperation of the NBDC in the Japan Science and Technology Agency (JST) since 2013. We have also provided a DDBJ group cloud service (https://www.ddbj.nig.ac.jp/dgc-e.html) that allows medical researchers to create a restricted data sharing service dedicated to personal genome data. In addition to those data archive services, the DDBJ has provided a login portal for personal data analysis on the top of the NIG supercomputer 2019 system. The system, including its calculation nodes, storage areas, networks, resource management system, etc. are independent of the other (general analysis) system and is protected by a series of dedicated security considerations. Those independent resources are provided on a per-project basis.

### The software system

The software system of the NIG supercomputer 2019 provides support for the management of a large variety of genome analysis tools that are prerequisites for large-scale genome analysis efforts. More than a thousand of those genome analysis tools are provided in the form of Singularity containers. Singularity is a Linux container system for HPC environments that can contain the tools together with their run-time environments, which allows them to absorb the run-time environment difference required for each tool (28). This feature makes it suitable for constructing a data analysis pipeline on the supercomputer and helps analysis reproducibility. The tools in the Singularity containers are executed with each user's execution authority, which also helps ensure security.

The task management system of the General Analysis Division (GAD) is provided by UGE. However, since the system of each project in the personal genome analysis division is independent of each other, user can choose other management systems, including the Slurm Workload Manager (https://slurm.schedmd.com/) and Kubernetes (https://kubernetes.io/).

### Integration with public clouds

The use of public cloud computing has been increasing rapidly. As an attempt to integrate supercomputers with the public cloud, the NIG supercomputer is connected to Amazon Web Services (AWS) by using the SINET5 network system hosted by the NII.

**Figure 2.**
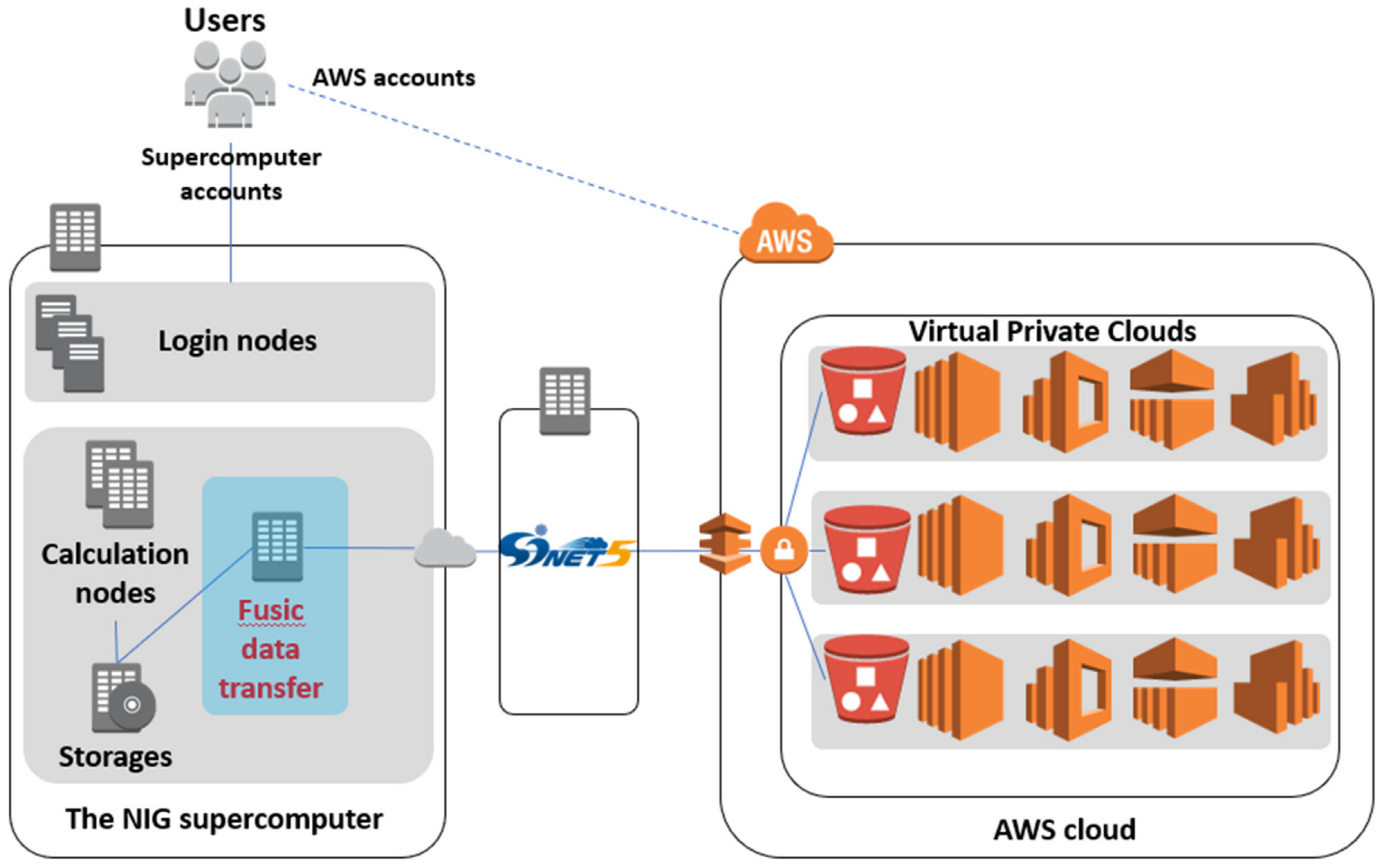
Automatic file transfer system between the NIG supercomputer and a public cloud (Amazon Web Service). Dedicated data transfer server (Fusic data transfer) is installed in the NIG supercomputer that allows users to send data, up and down compute instances, running jobs and make configuration changes on the AWS cloud by using a series of command line tools installed on the NIG supercomputer. SINET5 network is subject to discount for egress network traffic charge of the public cloud.

To facilitate this, we installed the Fusic data transfer system (https://fusic.co.jp/english/) on the NIG supercomputer (Figure 2). This allows users to construct computing instances on the AWS platform, transfer the data and programs from the supercomputer to the S3 storage, and perform computing instances on the AWS platform with a few command-line operations. Integration of user accounts is supported on a per-request basis.

## FUTURE DIRECTION

According to a recent report from analyst firm Forrester on behalf of Virtustream, the vast majority and increasing number of firms around the world characterized their cloud strategy as multi-cloud, which combines both on-premise and public-cloud resources, in order to avoid vendor lock-in, data lock-in and to achieve cost/performance balance by exploiting most suitable computing resources from among them (e.g. McLellan,C. (2019) Multicloud: Everything you need to know about the biggest trend in cloud computing., ZDNet. https://www.zdnet.com/article/multicloud-everything-you-need-to-know-about-the-biggest-trend-in-cloud-computing/). Toward this direction, we are planning to enhance the functionality of public cloud integration with supercomputers. Crucial functionalities for this purpose are deployment and resource management systems for the multi-clouds (29) and Kubernetes has been growing to be a prevailing cluster manager on both public-clouds and on-premise systems. On this basis, we are developing an inter-cloud infrastructure with the cooperation among National Institute of Information, Hokkaido University, Tokyo Institute of Technology, Kyusyu University, and National Institute of Genetics. We are also developing its underlining resource manager (30) with optimal cloud resource selection algorithms (31–33) and resource usage data gathering systems which is prerequisite of the optimization algorithms (34).

In order to accommodate the increasing demand for large-scale analysis on the NIG supercomputer system at present, we plan to install additional thin calculation nodes with about 3000 CPU cores in early 2020. These nodes will be used primarily in the GAD and are expected to mitigate any deficiency of calculation resources in our system substantially.
